# Assessing the reporting quality of physical activity programs in randomized controlled trials for the management of juvenile idiopathic arthritis using three standardized assessment tools

**DOI:** 10.1186/s12969-020-00434-9

**Published:** 2020-05-24

**Authors:** Teresa-Rose Kattackal, Sabrina Cavallo, Lucie Brosseau, Aditi Sivakumar, Michael J. Del Bel, Michelle Dorion, Erin Ueffing, Karine Toupin-April

**Affiliations:** 1 Faculty of Scienceskk, University of Ottawa, Ottawa, Ontario Canada; 2 School of Rehabilitation, Université de Montréal, Montréal, Québec Canada; 3 School of Rehabilitation Sciences, Faculty of Health Sciences, University of Ottawa, Ottawa, Ontario Canada; 4 Children’s Hospital of Eastern Ontario Research Institute, room L1147, 401 Smyth Road, Ottawa, ON K1H 8L1 Canada; 5 Department of Pediatrics, Faculty of Medicine, University of Ottawa, Ottawa, Ontario Canada

**Keywords:** Juvenile idiopathic arthritis, Physical activity programs, Randomized controlled trials, Reporting quality

## Abstract

**Background:**

The reporting quality of physical activity (PA) programs in randomized controlled trials (RCTs) for the management of juvenile idiopathic arthritis (JIA) remains unknown. This study aimed to assess and compare the reporting quality of PA programs in RCTs for the management of JIA using three difference standardized assessment tools, and to describe the elements that were similar and different between these tools.

**Methods:**

A systematic search was conducted for moderate-to high-quality RCTs of PA programs in JIA, published up until January 2019. Two reviewers independently included 10 RCTs and scored the reporting quality of PA programs using the following tools: Consensus on Exercise Reporting Template (CERT) checklist, Consensus on Therapeutic Exercise Training (CONTENT) scale, and Template for Intervention Description and Replication (TIDieR) checklist.

**Results:**

Results showed that reporting of PA programs in 10 moderate- to high-quality RCTs for JIA management remains incomplete. The average reporting quality (± standard deviation) for all RCTs combined was moderate for the three standardized assessment tools with 70.8 (±14.3)% for the TIDieR checklist, 53.2 (±20.2)% for the CERT checklist, and 70.0 (±18.9)% for the CONTENT scale. Despite some overlap, the three standardized assessment tools (TIDieR, CERT, CONTENT) included different elements resulting in different scores. All tools assess elements linked to PA programs (provider, location, timing, personalization and adherence), but the CERT checklist includes other essential elements (e.g., additional resources, motivational strategies, adverse events).

**Conclusions:**

The lack of complete reporting of PA programs in RCTs for the management of JIA and the variation in scores and assessed elements among standardized assessment tools show the need to improve reporting. Using the most comprehensive standardized tool (i.e.*,* the CERT) and providing accessible supplemental information on PA programs may improve the reporting quality of PA programs in RCTs and help reproduce PA programs in research and clinical practice.

## Background

Juvenile idiopathic arthritis (JIA) is a chronic childhood autoimmune disease, affecting approximately two in 1000 children [1]. JIA is characterized by prolonged inflammation in the youth’s joints including pain, redness and swelling [2], which often lead to physical, psychological and social limitations [3]. Practitioners diagnose JIA after symptoms are experienced in one or more joints for at least six consecutive weeks in youth under age 16 [4]. Such symptoms produce additional health and social difficulties: youth with JIA are less physically active than other kids, may miss school because of symptoms or appointments with health care providers, and may feel different from their peers when they cannot participate in physical education or play [3]. Healthy lifestyle choices, such as being physically active, are crucial in attaining improved outcomes in youth with JIA and as they become adults [5–8]. Moreover, children with JIA are developing their physical skills alongside their self-esteem and self-efficacy, which are crucial for their future [5, 7, 8]. It is particularly important for youth with JIA to learn how to self-manage their disease, which means that they should gain the knowledge and the ability to manage their symptoms, such as pain, and the consequences of their chronic condition on their life [7, 9]. This may be achieved by using various self-management strategies. In addition to pharmacological therapies and regimented exercises, treatment recommendations for JIA include recreational and physical activities (PA) within the child’s pain threshold for disease self-management [5].

PA programs are beneficial in various chronic diseases, such as fibromyalgia, chronic kidney disease, and heart failure [10], as well as JIA [7]. Specifically in JIA; the Ottawa Panel Evidence-Based Clinical Practice Guidelines [7], based on systematic reviews of randomized controlled trials (RCTs), found that most PA programs described improved JIA management. Their overall findings were that various PA programs, including pilates, home strengthening exercise programs, aquatic aerobic fitness programs, cardio-karate (aerobic exercise) and weight-bearing exercise programs improved various health outcomes such as pain, range of motion, muscle strength, quality of life and function [11–15]. The latest RCTs found that other PA programs, including balance-proprioceptive exercises, backward treadmill training, water-running programs, combination of resistive underwater exercises and interferential current therapy, combination of electromyographic biofeedback and a physical therapy program, also led to improved health outcomes such as balance, anaerobic exercise capacity, pain, muscle strength and function [16–20].

Although studies have shown that PA programs improve disease management and health outcomes, PA programs are not always well described [7]. This may be problematic since researchers and clinicians need specific details on PA programs in clinical trials to facilitate further high-quality research and to implement the most effective PA programs in clinical practice [21]. Since moderate- to high-quality RCTs frequently inform recommendations in clinical practice guidelines, it is important to assess the reporting quality of PA programs in these trials. The reporting quality of trials can be assessed using standardized tools, which could improve intervention descriptions in protocols and manuscripts, and allow for replication of PA programs in research and clinical practice [22]. According to the EQUATOR Network’s website and the scientific literature, three standardized tools can be used to assess the reporting quality of all types of PA programs in RCTs [23–26]. These tools are the Template for Intervention Description and Replication (TIDieR) checklist [23]; the Consensus on Exercise Reporting Template (CERT) checklist [24]; and the Consensus on Therapeutic Exercise Training (CONTENT) scale [25]. The TIDieR is applicable to most types of interventions [23] and is an extension of the CONSORT 2010 statement [27] and the SPIRIT 2013 statement [28]. The CERT is an extension of the TIDieR checklist, which provides more elements which are specific to exercise [24]. The CONTENT scale is another tool which is specific to exercise programs [25]. Other studies used these tools to assess the quality of reporting of PA programs in RCTs in other populations [29, 30].

The primary objective of this study was to assess the quality of reporting of PA programs in RCTs for the management of JIA using three different standardized assessment tools. The secondary objectives were: a) to compare the scoring of the three standardized assessment tools for all RCTs; and b) to describe the elements that are similar and different between the three standardized assessment tools.

## Methods

### Study design

First, we updated an earlier systematic search completed by Cavallo et al. [7], which identified moderate- to high-quality RCTs of PA programs for the management of JIA (including RCTs published up until May 2015). Second, we assessed the quality of reporting of PA programs in identified RCTs using three different standardized assessment tools.

### Search strategy

Our study applied a similar search strategy as Cavallo et al. [7] to identify moderate- to high-quality RCTs of PA programs for JIA management published between May 2015 and January 2019 using these electronic databases: Cochrane Central Register of Controlled Trials, EMBASE (Ovid) and MEDLINE (Ovid) (see Appendix 1). The search produced a list of records to be reviewed.

### Study selection

Two reviewers (T.K., A.S.) independently screened titles and abstracts of the records using pre-determined selection criteria, outlined by Cavallo et al. [7] Following the initial screening, the two independently assessed the eligible full-length articles to ensure all inclusion criteria were met. Finally, the two reviewers independently selected eligible articles and gained consensus. If consensus between the two reviewers was not reached, a third reviewer (M.D.B., L.B. or K.T.A.) facilitated a final decision. Eligible studies were RCTs of PA programs for JIA management, graded as moderate- to high-quality using the Physiotherapy Evidence Database (PEDro) scale (total scoring of 5 or greater out of 10 points). In addition, comparison groups needed to include a JIA population undergoing conventional therapy or lower intensity PA, or being on a wait list.

### Reviewers’ training with the three standardized assessment tools

Training on the three standardized assessment tools occurred prior to the scoring of all selected and eligible RCTs of PA programs for JIA management. Two junior reviewers (T.K., A.S.) were trained by a senior reviewer (M.D.B.) using a practice session with two published RCTs of PA programs for the management of fibromyalgia [31, 32]. The senior reviewer ensured that the scores between the two junior reviewers were consistent and that definitions of all items from each standardized assessment tool were understood.

### Study data extraction

An Excel spreadsheet was developed with the elements of the three standardized assessment tools and the characteristics of each RCT (e.g., year, authors’ names, types of PA interventions, control groups). If an RCT included two types of PA programs (i.e.*,* other than usual care), the reporting of the programs was assessed separately. After independently scoring the reporting quality of PA programs, the two reviewers (T.K., A.S.) compared individual item scores, for all RCTs, from each of the standardized assessment tools and came to a consensus. Each item from the three standardized assessment tools were scored as 1 (reported) or 0 (not reported). A third senior reviewer (M.D.B) served as an arbitrator if needed for disagreements. E-mails were sent to authors to ask for additional information on their PA programs, including supplementary material, with up to two reminders.

### Reporting quality of physical activity programs in randomized controlled trials

Three standardized assessment tools were used for assessing the reporting quality: Template for Intervention Description and Replication (TIDieR) checklist [23]; Consensus on Exercise Reporting Template (CERT) checklist [24]; and Consensus on Therapeutic Exercise Training (CONTENT) scale [25].

### TIDieR checklist

The TIDieR checklist and guide (Table 2 in Appendix 2) were developed based on a literature review, a Delphi survey of international experts, and a panel meeting, with the aim of providing authors the minimum amount of information required for describing an intervention [23]. The TIDieR checklist was found to be applicable to most types of interventions, including pharmacological interventions, with the expectation that authors would provide additional information when required for their specific intervention type [23]. It is an extension of the CONSORT 2010 statement [27] and the SPIRIT 2013 statement [28]. The TIDieR checklist has 12 items: brief name of the intervention; goal of the intervention; materials used in the intervention; procedure; provider; how the intervention was delivered; where the intervention was delivered (location); when the intervention was delivered; how much of the intervention was delivered (dosage); tailoring of the intervention to each individual; modifications made to the intervention; whether the intervention was well-planned; and well-delivered.

### CERT checklist

The CERT checklist (Table 3 in Appendix 2) was developed using the EQUATOR Network’s methodological framework through a meta-epidemiologic study, a Delphi survey and a Delphi workshop, with the aim of providing authors direction for reporting exercise interventions by including key items that are considered essential for replicating of exercise program interventions [24]. This checklist is an extension of the TIDieR checklist, providing more assessed elements and reporting requirements [24]. Similar to the TIDieR checklist, there is the expectation that reporting additional information may be required depending on the type of exercise program. The CERT checklist has 19 items covering seven categories: materials used in the intervention; provider; how the intervention was delivered; where the intervention was delivered; when the intervention was delivered/how much of the intervention was delivered (dosage); tailoring of the intervention to each individual; and how well participants followed the intervention (compliance).

### CONTENT scale

The CONTENT scale (Table 4 in Appendix 2) was created based on consensus findings from a Delphi study and was designed to determine the therapeutic effectiveness of an exercise program (evaluated based on an individual score of 6 out of 9 CONTENT scale items or higher) [25]. This scale guides authors in providing sufficient reporting detail for readers to understand how the intervention was implemented. The CONTENT scale is a 9-item scale that contains 17 sub-items. There are five specific item categories: patient eligibility; competences of the provider and setting of the exercise program; rationale; content; and adherence to the exercise program.

### Descriptive statistical analysis

#### Standardized assessment tools’ scores for all RCTs combined

For each standardized assessment tool, the total mean scores and standard deviation (SD) were calculated using the individual RCT scores. A 95% confidence interval was also computed for each standardized assessment tool. To compare the total mean scores of each standardized assessment tool, the total mean scores were expressed as a percentage. The reporting quality of the RCTs was evaluated by each standardized assessment tool using this grading method for each mean percentage score: 50% or less = poor; 51 to 79% = moderate; and 80 to 100% = good, as described by Mack et al. [33]

#### Standardized assessment tools’ scores for individual RCTs

To assess the reporting quality, each PA program in RCTs for JIA received three individual scores, one from each of the three standardized assessment tools. The three individual scores were reported as scores out of: 12 (TIDieR checklist), 19 (CERT checklist) and nine (CONTENT scale). Subsequently, the individual scores were reported as a percentage score, thus each PA program in RCTs for JIA received three percentage scores.

#### Reporting of each item from the three standardized assessment tools

At the level of each standardized assessment tool, the reporting of each item was expressed as a percentage of RCTs which reported this item. A descriptive analysis was used to identify the items of each standardized assessment tool which were most and least reported.

#### Shared and different elements between the three standardized assessment tools

The elements that were shared between all three tools were noted, along with those that were different. Items were categorized across tools as overlap, uncertain overlap or no overlap.

## Results

### Search results

**Fig. 1:**
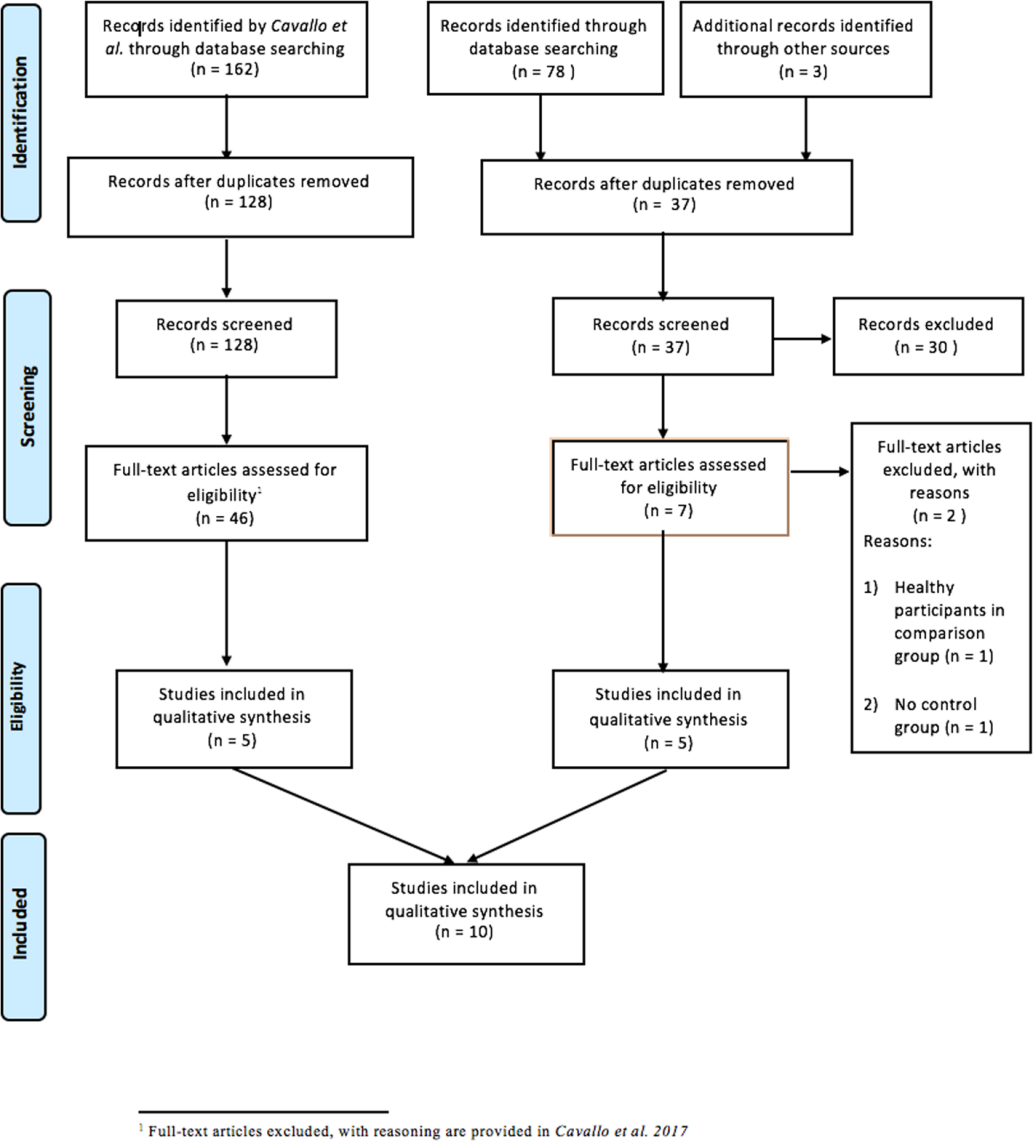
Study flow diagram (Preferred Reporting Items for Systematic Reviews and Meta-Analysis (PRISMA)) for selected RCTs. This PRISMA flow chart is developed using the “PRISMA Statement” referenced in: *Moher D, Liberati A, Tetzlaff J, Altman DG, The PRISMA Group (2009) Preferred Reporting Items for Systematic Reviews and Meta-Analyses: The PRISMA Statement. PLoS Med 6* (7)*: e1000097.*10.1371/journal.pmed.1000097

The update of the systematic search (from 2015 to present) produced 37 articles after removing duplicates. Of those, five were included after two selection rounds [16–20]. These were added to the five RCTs found by Cavallo et al. [7] in their previous search (until 2015) [11–15], totaling10 RCTs (Fig. 1). Four of the authors replied, but none provided additional information on their PA programs. Five of the RCTs had supplementary material, but we could access it only for one RCT [11], and thus did not consider this information to treat all RCTs equally.

### Standardized assessment tools’ scores for all RCTs combined

The average reporting quality for all RCTs combined was moderate for the three standardized assessment tools. The mean percentage (± standard deviation) total reporting quality score for RCTs evaluated was 70.8 (±14.3) % for the TIDieR checklist, 53.2 (±20.2) % for the CERT checklist, and 70.0 (±18.9) % for the CONTENT scale (Table 1).

**Table 1:** Individual, total and percentage consensus scores for the TIDieR checklist, the CERT checklist, and the CONTENT scale

Author / Year	TIDieR Score out of 12 (%)	CERT Score out of 19 (%)	CONTENT Score out of 9 (%)	Scoring Variation (%)
Baydogan (2015) [16]	8 (66.7)	11 (57.9)	6 (66.7)	8.8
Bayraktar (2019) [18]	11 (91.6)	13 (68.4)	8 (88.9)	23.2
Eid (2016) [20]	6 (50.0)	6 (31.6)	4 (44.5)	18.4
Elnaggar (2016) [19]	9 (75.0)	8 (42.1)	6 (66.7)	32.9
El Aziz (2017) [17]	7 (58.3)	5 (26.3)	5 (55.6)	32
Mendonca (2013) [11]	10 (83.3)	16 (84.2)	7 (77.8)	6.4
Sandstedt (2013) [15]	8 (66.7)	7 (36.8)	4 (44.5)	29.9
Singh-Grewal (2007) [14]	8 (66.7)	12 (63.2)	8 (88.9)	25.7
Takken (2003) [13]	7 (58.3)	8 (42.1)	6 (66.7)	24.6
Tarakci (2012) [12]	11 (91.6)	15 (78.9)	9 (100.0)	21.1
**Total mean score (SD)**	8.5 (± 1.7)	10.1 (± 3.8)	6.3 (± 1.7)	________
**Total mean percentage (SD) (%)**	70.8 (±14.3)	53.2 (±20.2)	70.0 (±18.9)	22.3 (±9.0)

### Standardized assessment tools’ scores for individual RCTs

None of the RCTs had complete reporting quality for PA programs. The highest scoring RCTs for the TIDieR checklist, CERT checklist, and CONTENT scale were: Bayraktar et al. [18] and Tarakci et al. [12]; Mendonca et al. [11]; and Tarakci et al. [12], respectively. The lowest scoring RCTs for the TIDieR checklist, CERT checklist, and CONTENT scale were: Eid et al. [20]; El Aziz et al. [17]; and Eid et al. [20] and Sandstedt et al. [15], respectively (Table 1).

### Reporting of each item from the three standardized assessment tools

#### TIDieR checklist

**Fig. 2:**
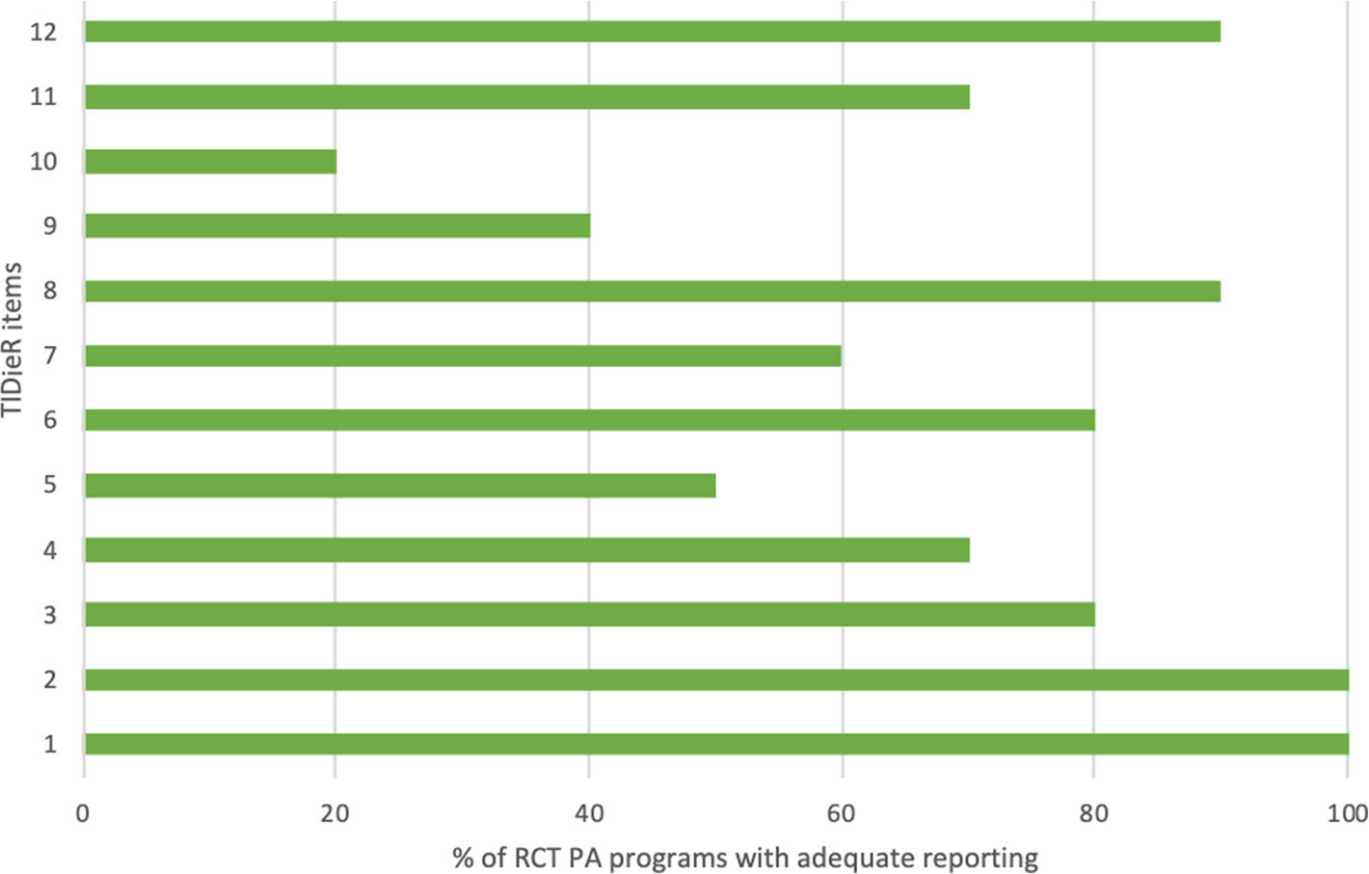
Percentage of RCTs (*n* = 10) which reported each TIDieR checklist item (see Appendix 2: item descriptions)

The highest score was 11 out of 12, which was given to two studies [12, 18], while the lowest was 6 out of 12 [17, 20] (Table 1). The majority of RCTs met the TIDieR criteria for item 1 (brief name of the intervention) and item 2 (rationale of the intervention). In contrast, most of the RCTs did not satisfy item 9 (tailoring of the intervention to each individual) and item 10 (modifications made to the intervention) (Fig. 2).

#### CERT checklist

**Fig. 3:**
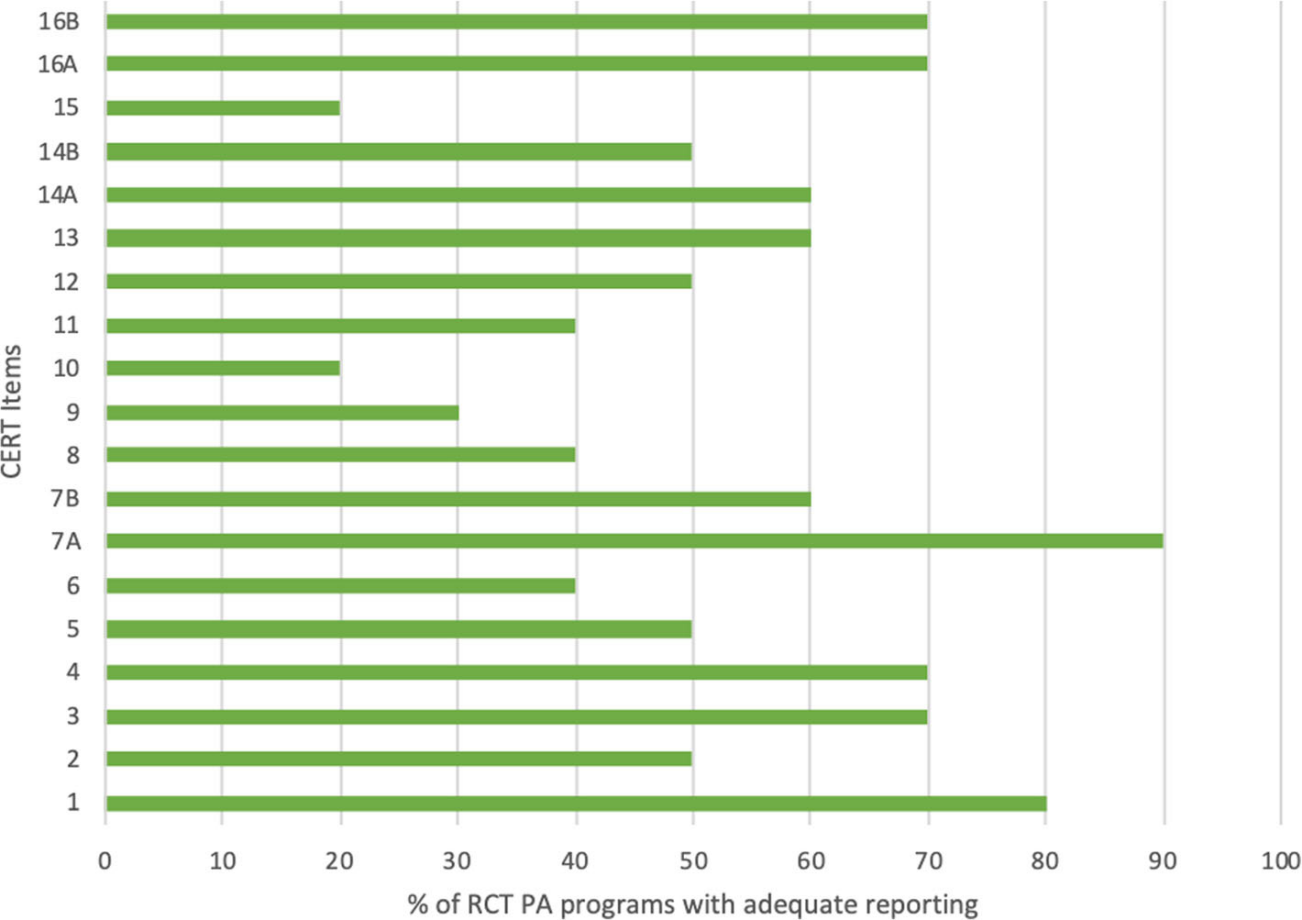
Percentage of RCTs (*n* = 10) which reported each CERT checklist item (see Appendix 2: item descriptions)

The highest score was 16 out of 19 [11], while the lowest score was 6 out of 19 [20] (Table 1). Most RCTs satisfied these items: item 1 (necessary exercise equipment), item 3 (exercise performed individually or in a group), and item 7A (way in which to progress through the exercise program). Most RCTs did not satisfy these items: item 6 (motivation strategies), item 8 (exercise description), item 9 (at home program component), item 10 (non-exercise components), item 11 (adverse events) and item 15 (decision rule for starting the level of the exercise) (Fig. 3).

#### CONTENT scale

**Fig. 4:**
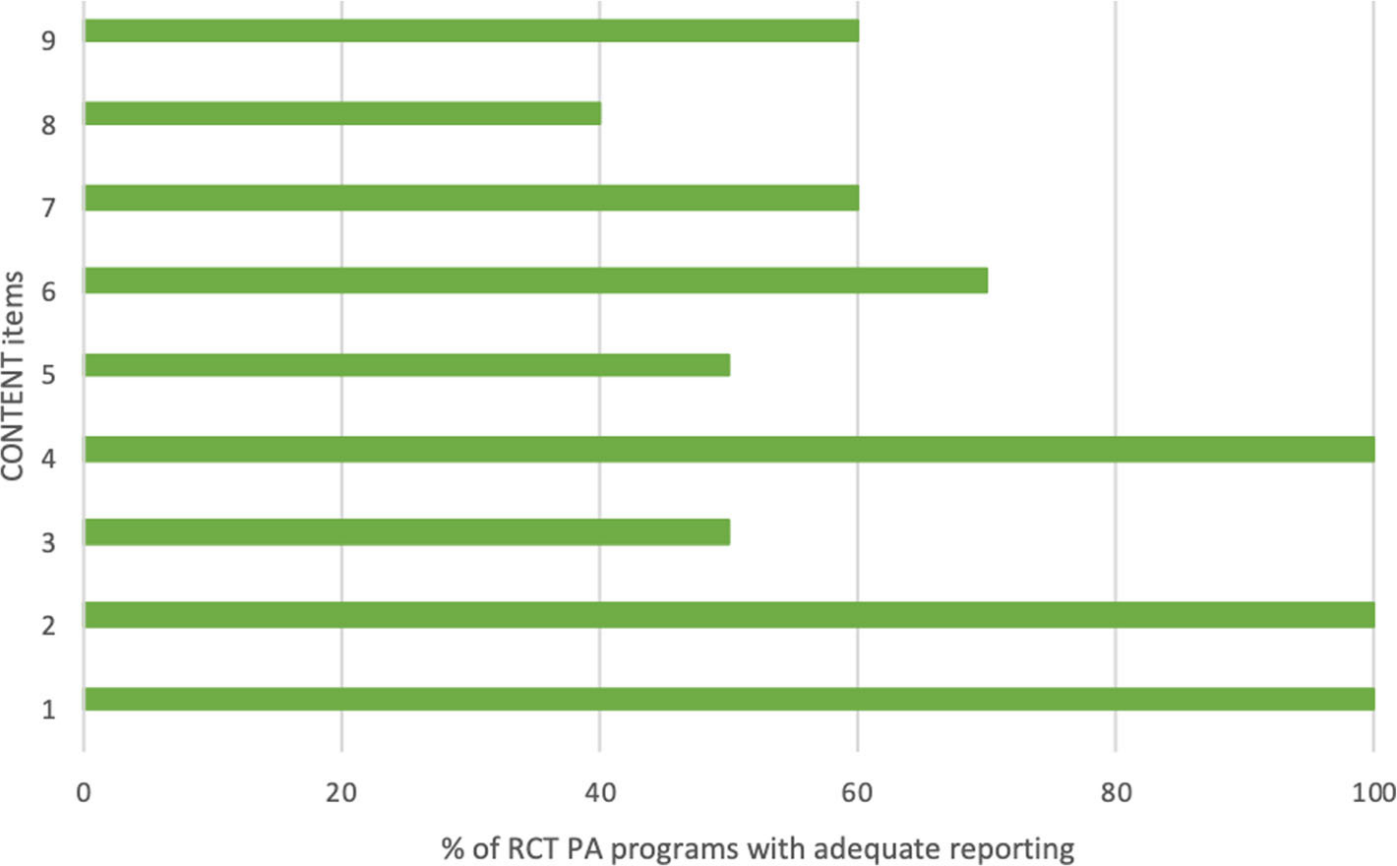
Percentage of RCTs (*n* = 10) which reported each CONTENT scale item (see Appendix 2: item descriptions)

The highest score was 9 out of 9 [12] while the lowest score was 4 out of 9 [15] (Table 1). Of the nine items from the CONTENT scale, most RCTs reported item 1 (how patients were selected), item 2 (whether the selection of patients was adequate), and item 4 (a priori aims and intentions of the program). Most of the RCTs did not satisfy item 8 (whether therapeutic exercises are personalized or contextualized) (Fig. 4).

### Shared and different elements between the three standardized assessment tools

None of the checklists had a perfect overlap of items (Appendix 3). The shared elements between the three standardized assessment tools were: (1) who provided the intervention along with their qualification; (2) where was the intervention taking place (setting); (3) when was the intervention held and how frequently (intensity); (4) personalization of the intervention to a given participant; (5) any modifications to the intervention; and (6) the adherence to the intervention. An element only required by the TIDieR checklist was a name or phrase that describes the intervention (brief name). Elements only required by the CERT checklist were: motivational strategies used, a detailed description to enable replication (including additional resources for researchers); a description of at home components; and the types and number of adverse events. Elements only required by the CONTENT scale were a description of patient selection and a description of the adequacy of patient selection.

## Discussion

Overall, the quality of reporting was moderate for the PA programs [11–20] in 10 moderate- to high-quality RCTs for the management of JIA, based on three standardized assessment tools. Scores varied among the three tools. Six elements were shared among the three tools: who provided the intervention; the location of the intervention; when and how often the intervention was delivered; personalization of the intervention for participants; modifications made to the intervention; and overall adherence to the program. Our findings of moderate reporting quality of PA programs in RCTs for JIA management are consistent with a study on knee osteoarthritis management when using the CERT checklist and the American College of Sports Medicine guideline [34]. In addition, studies in fibromyalgia management and stroke rehabilitation had consistent results of moderate reporting quality of PA programs in RCTs when using the TIDieR checklist, CERT checklist, and CONTENT scale [29, 30].

The moderate reporting quality of PA programs in RCTs for JIA management may be explained by the lack of requirements by journals for authors to use reporting guidelines, and journal restrictions on length of manuscripts and supplementary materials [22, 35]. Concerning the use of reporting guidelines, Turner et al. [36] compared the reporting quality of RCTs published in journals which endorsed the CONSORT statement (a general reporting tool for RCTs) with those which did not, and found that journals which endorsed the CONSORT statement had overall greater likelihood of more complete reporting. This suggests that reporting quality of RCTs across various disciplines could be improved through the endorsement of reporting tools by journals [35]. Concerning the restrictions on length of manuscripts and supplementary material, our results showed that RCTs with the highest reporting quality generally included more figures, tables, flow-charts and/or lists [11, 12, 16]. Also, two of these RCTs were similarly scored between the three standardized assessment tools [11, 16]. Including further detail through concise means (e.g. figures, tables) allowed authors to meet the word count and page limit while including important reporting details. Supplementary material may be useful, but these should be easily accessible, which was not the case in our search for information. In fact, Abell et al. [37] noted that the publication of supplemental material increases reporting completeness of exercise interventions in RCTs for cardiac rehabilitation from 8 to 43% [37]. Through the guidance of reporting tools, the use of more concise reporting means, and the publication of easily accessible supplemental material, such as instructional videos, images, tables and protocol papers, authors may overcome manuscript length restrictions and allow for adequate reporting.

Concerning the mean total reporting scores for the three standardized assessment tools, all three were of moderate quality, with the CERT checklist having the lowest score (53.2%) compared to the TIDieR checklist (70.8%) and the CONTENT scale (70.0%). The moderate overall reporting quality for the PA programs may be explained by the fact that the three tools share six key elements, which indicate a level of similarity in some of the core reporting requirements. In general, elements such as the dosage of the exercise or PA program, and the material used were better reported than information on the intervention progression or tailoring, the adherence, any noted adverse events or the motivational strategies used.

Two elements which were shared between the three tools and were under-reported were: (1) qualification level of those who lead the intervention and whether the exercises were supervised or unsupervised; and (2) modifications (or lack thereof) made to the intervention. These elements were also under-reported in hypertension management, physiotherapy, and educational studies [38–41]. Particular importance should be given to the lack of information about modification and fidelity of PA programs (Items 10–12 of the TIDieR checklist). Reporting on modification and fidelity ensures the adequate assessment by knowledge-users of the interventions’ limitations and feasibility of implementation in clinical practice [42].

The low score for the CERT may be explained by the fact that items which were the least reported (with 50% or less RCTs meeting these reporting requirements) were unique to the CERT. These unique elements with low scores include: description to enable replication (must include resources), description of at home components, motivational strategies used and the type and number of adverse events. Similar results of incomplete reporting of these elements were seen in other studies which assessed the reporting quality of PA programs using CERT in RCTs for cerebral palsy and hypertension [38, 43]. This may be explained by the fact that the CERT checklist contains a greater level of detail and more items describing elements related to the procedure and execution of exercise interventions than the other standardized tools. It is important to note that a number of elements were included in the CERT checklist since it was developed specifically to enhance the reporting quality of exercise programs, and acts as an extension of the TIDieR checklist applicable to any intervention type [44, 45]. The elements unique to the CERT checklist are essential to assess the reporting quality of PA programs in trials, according to experts involved in a rigorous process [24]. In particular, the details of adherence of the participant (item 5) and the service provider (item 16a), allow an explicit description of patient adherence to the agreed intervention plan, as well as fidelity of delivering and performing the intervention as planned by the developer [24, 38]. Also, motivational strategies increase the effectiveness of exercise [24]. Unlike the CERT, the TIDieR only identifies potential personal motivating factors helping to respond to the individual’s context without assessing comprehensive solutions [23]. These elements provide much needed information to better understand the limitations in service provision and implementation. Further, these findings highlight the importance of considering the different levels of specificity and comprehensiveness of these tools (i.e.*,* CERT for exercise programs vs. TIDieR for any type of intervention).

Other elements that are of interest include those focusing on the selection of participants which are unique to the CONTENT scale. Interestingly, these elements are also part of the CONSORT statement, which is recommended in addition to the TIDieR and CERT checklists when assessing RCT quality.

Overall, the CERT checklist is the most comprehensive to evaluate the reporting quality of a wide range of PA programs in RCTs. Since its total reporting quality score for RCTs was relatively low, efforts should be made to improve reporting of its various elements, especially the description of the PA programs to enable replication (with resources such as accessible instructional videos, images, tables and protocol papers), description of at home components, motivational strategies used and the type and number of adverse events.

### Limitations

Selection and information bias are possible in this study due to a small sample size (*n* = 10) and the possibility of missing relevant studies (e.g., published in other languages). RCTs were selected through a systematic search of three online databases and the criteria of being moderate- to high-quality trials based on PEDro standards [46]. Including lower quality trials may have resulted in lower quality results and possibly overall lower quality reporting in RCTs. In addition, a larger sample size would allow for the analysis of other trends (e.g., publication date).

Inclusion of supplemental information from authors, during the process of assessing content reporting, may have increased the overall reporting quality in the given sample [37]. Although this process may help to improve completeness of the information, it remains lengthy and difficult since this information is not always easily accessible. In addition, as this study aimed to diminish inter-rater variation, consensus of scores was gained between reviewers, without the help of a senior reviewer.

## Conclusions

In summary, results showed that reporting of PA programs in 10 moderate- to high-quality RCTs for JIA management remains incomplete. Despite some overlap, the three standardized assessment tools (TIDieR, CERT, CONTENT) included different elements resulting in different scores, which highlights the need to choose the most suitable standardized tool to report PA programs in RCTs. Using the most comprehensive standardized tool (i.e.*,* the CERT) and providing accessible supplemental information on PA programs may improve the reporting quality of PA programs in RCTs and help reproduce PA programs in research and clinical practice.

### Recommendations for future RCTs reporting PA programs for the management of JIA

Future studies may improve the reporting quality of PA programs in RCTs by: (1) using the most comprehensive standardized tool to report PA programs; and (2) providing accessible supplemental information for clinicians to help reproduce PA programs in research and clinical practice.

### Clinical messages

- Reporting of PA programs in moderate-to high-quality RCTs for JIA management remains incomplete.
- The three reporting tools included different elements and gave different scores, which highlights the need to choose the most comprehensive standardized tool to report PA programs in RCTs.
- Accessible instructional videos, images, tables and protocol papers, as well as the use of reporting tools may help improve information completeness and subsequent reporting quality which could possibly improve clinical implementation.

## Figures and Tables

**Fig. 5:**
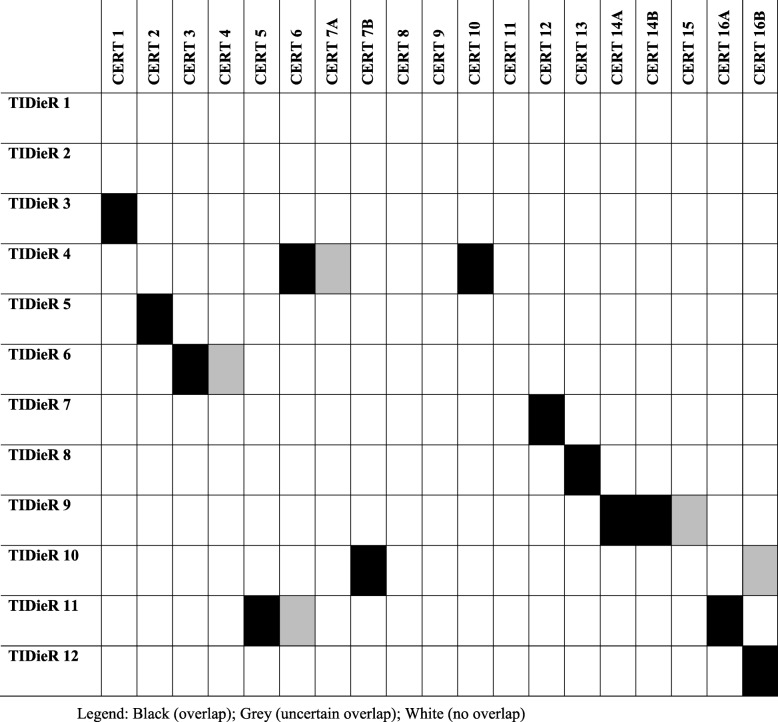
Overlap of items between the CERT and TIDieR checklists (see Appendix 2 for item descriptions). Legend: Black (overlap); Grey (uncertain overlap); White (no overlap)

**Fig. 6:**
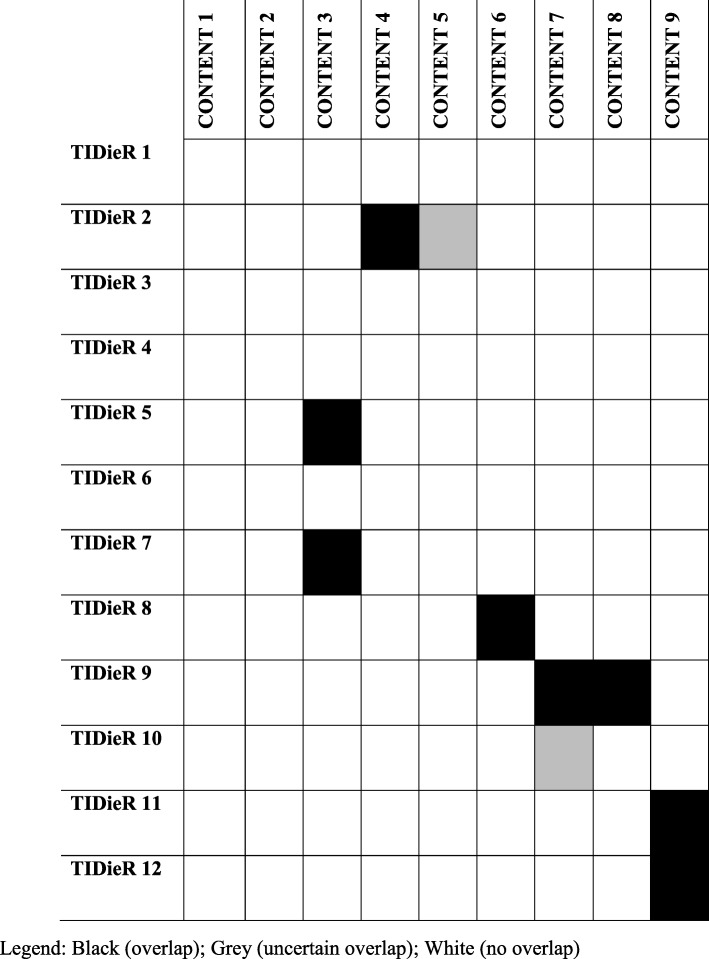
Overlap of items between CONTENT scale and TIDieR checklist (see Appendix 2 for item descriptions). Legend: Black (overlap); Grey (uncertain overlap); White (no overlap)

**Fig. 7:**
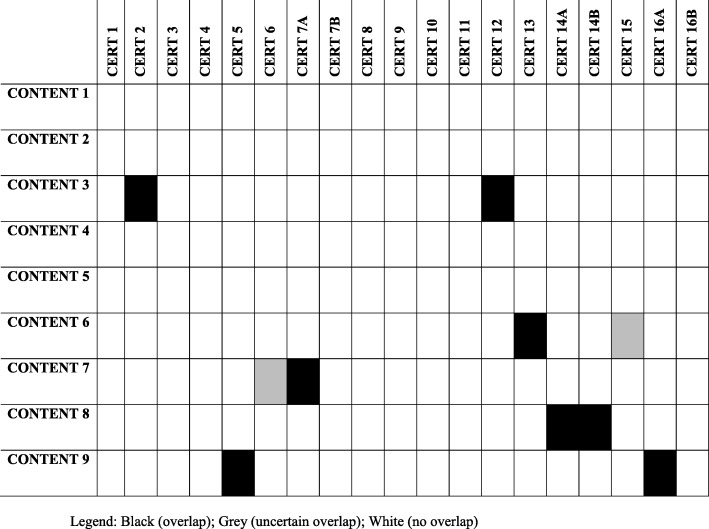
Overlap of items between CERT checklist and CONTENT scale (see Appendix 2 for item descriptions). Legend: Black (overlap); Grey (uncertain overlap); White (no overlap)

## Data Availability

Not applicable.

## Appendix 1

### MEDLINE (OVID) search strategy

1. arthritis, juvenile/

2. arthritis, psoriatic/

3. 1 or 2

4. (juvenile adj2 arthritis).tw.

5. 3 or 4

6. clinical trial.pt.

7. randomized controlled trial.pt.

8. random$.tw.

9. (double adj blind$).tw.

10. placebo$.tw.

11. meta-analysis.pt.,sh.

12. (meta-anal: or metaanal:).tw.

13. (quantitativ: review: or quantitativ: overview:).tw.

14. (methodologic: review: or systematic: overview:).tw.

15. (systematic: review: or systematic: overview:).tw.

16. review.pt. and medline.tw.

17. exp. cohort studies/

18. (cohort or longitudinal or prospective).tw.

19. exp. case-control studies/

20. (retrospective or case-control).tw.

21. Controlled Clinical Trial/

22. (controlled adj2 trial$).tw.

23. or/6–22.

24. therap$ exercise$.tw.

25. exp. exercise therapy/

26. (passive adj2 exercis$).tw.

27. mobilizing exercis$.tw.

28. ((strength$ or resistance or aerobic) adj exercis$).tw.

29. (continuous passive motion or movement device).tw.

30. exp. exercise/

31. exp. sports/

32. exp. exercise movement techniques/

33. (sport* or aqua* or swim*).tw.

34. (Taichi or “tai chi” or taiji or “tai ji” or yoga or pilates).tw.

35. or/24–34

36. plyometric exercise/ or plyometric.tw.

37. 35 not 36

38. manual therap$.tw.

39. exp. manipulation orthopedic/

40. (manipulation adj (therap$ or joint)).tw.

41. mobilization.tw.

42. or/38–41

43. 5 and 23

44. 37 or 42

45. 43 and 44

46. (2015 05* or 2015 06* or 2015 07* or 2015 08* or 2015 09* or 2015 10* or 2015 11* or 2015 12*).dt.

47. (2016* or 2017* or 2018* or 2019*).dt.

48. 46 or 47

49. 45 and 48

## Appendix 2

**Table 2:** Individual RCT TIDieR checklist scores based on reviewer consensus

Author (Year)	TIDieR Items											
	1	2	3	4	5	6	7	8	9	10	11	12
	BRIEF NAME	WHY	WHAT: Materials	WHAT: Procedure	WHO: Intervention provider	HOW: Mode of delivery	WHERE	WHEN, HOW: Duration	TAILORING/ PROGRESSION	MODIFICATION	HOW WELL: Measure of adherence	HOW WELL: Actual adherence
Baydogan (2015) [16]	1	1	1	1	1	1	0	1	0	1	0	1
Bayraktar (2019) [18]	1	1	1	1	0	1	1	1	1	1	1	1
Eid (2016) [20]	1	1	1	1	0	0	0	1	0	0	0	1
Elnaggar (2016) [19]	1	1	1	1	0	1	1	1	0	0	1	1
El Aziz (2017) [17]	1	1	0	1	0	0	1	1	1	0	0	0
Mendonca (2013) [11]	1	1	1	1	1	1	0	1	1	0	1	1
Sandstedt (2013) [15]	1	1	1	0	0	1	1	1	0	0	1	1
Singh-Grewal (2007) [14]	1	1	1	0	1	1	0	1	0	0	1	1
Takken (2003) [13]	1	1	0	0	1	1	1	0	0	0	1	1
Tarakci (2012) [12]	1	1	1	1	1	1	1	1	1	0	1	1

0: item is not reported; 1: item is reported

**Table 3:** Individual RCT CERT checklist scores based on reviewer consensus

Author (Year)	CERT Items																		
	1	2	3	4	5	6	7A	7B	8	9	10	11	12	13	14A	14B	15	16A	16B
	WHAT: Materials	WHO: Intervention provider	HOW: Individually or group	HOW: Supervised	HOW: Measure of adherence	HOW: Motivation strategy	HOW: Progression through program	HOW: Progressions	HOW: Exercise description	HOW: Home program component	HOW: Non-exercise components	HOW: Adverse events	WHERE: Location	WHEN, HOW MUCH	TAILORING: Generic or Individual	TAILORING: Individual tailoring	TAILORING: Starting level	HOW WELL: Adherence to intervention	HOW WELL: Delivered as planned
Baydogan (2015) [16]	1	1	1	1	0	0	1	1	1	1	0	0	0	1	1	1	0	0	0
Bayraktar (2019) [18]	1	0	1	0	1	1	1	1	0	0	0	1	1	1	1	1	1	1	1
Eid (2016) [20]	1	0	0	0	0	0	1	1	0	0	0	0	0	1	1	1	0	0	0
Elnaggar (2016) [19]	0	0	1	1	0	0	1	0	0	0	1	1	1	0	0	0	0	1	1
El Aziz (2017) [17]	1	0	0	0	0	0	1	1	0	0	0	0	1	0	0	1	0	0	0
Mendonca (2013) [11]	1	1	1	1	1	1	1	1	1	0	0	1	0	1	1	1	1	1	1
Sandstedt (2013) [15]	1	0	0	1	1	0	0	0	1	0	0	0	0	0	1	0	0	1	1
Singh-Grewal (2007) [14]	0	1	1	1	1	1	1	0	1	1	0	1	0	1	0	0	0	1	1
Takken (2003) [13]	1	1	1	1	0	0	1	0	0	0	0	0	1	0	0	0	0	1	1
Tarakci (2012) [12]	1	1	1	1	1	1	1	1	0	1	1	0	1	1	1	0	0	1	1

0: item is not reported; 1: item is reported

**Table 4:** Individual RCT CONTENT scale scores based on reviewer consensus

Author (Year)	CONTENT Items								
	1	2	3	4	5	6	7	8	9
	WHO: Patient selection	WHO: Adequate patient selection	WHO, WHERE: Therapist and setting	WHY: A-priori aims	WHY: Content and intensity rationale	WHAT: Intensity	HOW: Monitored and adjusted	HOW: Personalized and contextualized	HOW: Adherence
Baydogan (2015) [16]	1	1	1	1	0	1	1	0	0
Bayraktar (2019) [18]	1	1	0	1	1	1	1	1	1
Eid (2016) [20]	1	1	0	1	0	1	0	0	0
Elnaggar (2016) [19]	1	1	0	1	0	1	1	0	1
El Aziz (2017) [17]	1	1	0	1	1	1	0	0	0
Mendonca (2013) [11]	1	1	0	1	0	1	1	1	1
Sandstedt (2013) [15]	1	1	1	1	0	0	0	0	0
Singh-Grewal (2007) [14]	1	1	1	1	1	0	1	1	1
Takken (2003) [13]	1	1	1	1	1	0	0	0	1
Tarakci (2012) [12]	1	1	1	1	1	1	1	1	1

0: item is not reported; 1: item is reported

## Abbreviations

CERT: Consensus on Exercise Reporting Template
CONSORT: Consolidated Standards of Reporting Trials
CONTENT: Consensus on Therapeutic Exercise Training
EQUATOR network: Enhancing the QUAlity and Transparency Of health Research
JIA: Juvenile idiopathic arthritis
PA: Physical activity
PEDro: Physiotherapy Evidence Database
RCTs: Randomized controlled trials
SD: Standard deviation
TIDieR: Template for Intervention Description and Replication
